# Potential for identification of wild night-flying moths by remote infrared microscopy

**DOI:** 10.1098/rsif.2022.0256

**Published:** 2022-06-22

**Authors:** Meng Li, Clara Seinsche, Samuel Jansson, Julio Hernandez, Jadranka Rota, Eric Warrant, Mikkel Brydegaard

**Affiliations:** ^1^ Department of Physics, Lund University, Sölvegatan 14c, 22363 Lund, Sweden; ^2^ Department of Biology, Lund University, Sölvegatan 35, 22362 Lund, Sweden; ^3^ Department of Biology, University of Cologne, Zuelpicher Straße 47b, 50931 Cologne, Germany; ^4^ FaunaPhotonics, Støberigade 14, 2450 Copenhagen, Denmark; ^5^ Norsk Elektro Optikk A/S, Østensjøveien 34, 0667 Oslo, Norway; ^6^ Biological Museum, Department of Biology, Lund University, Sölvegatan 37, 22362 Lund, Sweden

**Keywords:** Lepidoptera, remote sensing, microstructures, surface roughness, infrared spectroscopy, hyperspectral imaging

## Abstract

There are hundreds of thousands of moth species with crucial ecological roles that are often obscured by their nocturnal lifestyles. The pigmentation and appearance of moths are dominated by cryptic diffuse shades of brown. In this study, 82 specimens representing 26 moth species were analysed using infrared polarimetric hyperspectral imaging in the range of 0.95–2.5 µm. Contrary to previous studies, we demonstrate that since infrared light does not resolve the surface roughness, wings appear glossy and specular at longer wavelengths. Such properties provide unique reflectance spectra between species. The reflectance of the majority of our species could be explained by comprehensive models, and a complete parametrization of the spectral, polarimetric and angular optical properties was reduced to just 11 parameters with physical units. These parameters are complementary and, compared with the within-species variation, were significantly distinct between species. Counterintuitively to the aperture-limited resolution criterion, we could deduce microscopic features along the surface from their infrared properties. These features were confirmed by electron microscopy. Finally, we show how our findings could greatly enhance opportunities for remote identification of free-flying moth species, and we hypothesize that such flat specular wing targets could be expected to be sensed over considerable distances.

## 1. Introduction

The lives of moths (order Lepidoptera) are obscure for most people owing to their nocturnal lifestyles. Most of our encounters with these insects are limited to their erratic fluttering flights around our light bulbs at night [1], and enthusiasts and biologists attempt to understand their behaviour and diversity using moth light traps and illuminated sheets [2]. There are hundreds of thousands of species of moths—10-fold the number of butterfly species [3–5]—and include infamous agricultural pest species such as army worms [6], corn- and sugarcane borers [7] and cutworms [8], all of which are pests with an enormous potential to inflict severe economic damage [9]. Other species are beneficial, such as the silkworm moth, the edible mopane worm (the larva of a species of emperor moth) and the Bogong moth, an iconic species of immense importance to the health of Australian alpine ecosystems [10–12]. While we sleep, moths take the night shift of pollinating our crops [13], and they provide a source of food that sustains a diverse group of predators [14]. Moreover, migratory moths transport megatons of biomass worldwide, providing a rich source of energy and nutrition for a variety of animals along the way [15].

How might we gain more insight into the fascinating lives and natural behaviours of moths? The disciplines of animal telemetry [16] and entomological radar [17] have made great progress in our understanding of moth behaviour over recent decades, and harmonic radar has been shown to track moth movements in the landscape [18], although the sample size is small in such tagging studies. The impressive dispersals of nocturnal insects have been visualized by continental networks of meteorological Doppler radars [19], with vertical polarimetric radar capable of tracking enormous numbers of untagged species above the ground clutter altitude [20]. The radar signal reflects the liquid water distribution in the moths, and the observations can be grouped by size [21], elongation [22] and, to some extent, by wingbeat frequency [23]. Despite these advances, it has not been possible to identify moths at the level of families and species with microwaves and radar.

At first glance, the coloration of moths is dull and dominated by different degrees of melanin [24,25]. Although some species display warning colours and mimic predators' eyes (e.g. the owl moth), such markings often constitute a minor fraction of the entire wings. Such markings also generally appear on the dorsal side of the wings, meaning that ground-based remote sensing technology has a limited ability to probe such spectral features [26]. The ventral sides of moth wings exhibit shades of brown [27], which is somewhat discouraging in terms of the potential for differentiating moth species remotely. The scattering phase function (the so-called bidirectional reflectance distribution function (BRDF)) recorded from the majority of moths displays a diffuse and Lambertian behaviour [28]. In other words, the ventral sides of moth wings are seldom glossy, and thus their oscillatory scattering cross-section waveform during a wingbeat does not differ more than their wingbeat dynamics [29] because of their wing shape projection throughout the wingbeat [30]. This waveform can be effectively described by only three harmonics, while a richer parameter space would yield a larger opportunity for identifying individual species.

Visual appearance and crypsis are not the only functions that could drive the evolution of wing surface morphology in moths. Other functions might include optimization of aerodynamic [31] or hydrophobic properties [32] or minimizing ultrasonic reflectance in relation to bat predation [33]. Moreover, some species possess androconial scales that produce species-specific pheromones used for mate attraction [34], while in the dot-underwing moth sexually dimorphic wing coloration might be used as a sexual signal during courtship [35]. Thus, scale morphology may also be associated with sexual selection.

While butterflies have fascinated scientists for centuries with their ability to manipulate daylight through organized nanostructures or photonic crystals [36–40], nocturnal moths have a limited benefit for such manipulations. Similarly, the relation between pigmentation and thermoregulation would therefore have little influence on the absorption of incoming visible daylight for the moth. Since thermoregulation is based on the incoming and outgoing energy balance, this leads to an intriguing thought: since butterflies manipulate visible reflectance by nanostructures, the organization of microstructures in moths might govern the thermal infrared emissivity spectrum associated with radiative cooling [41], leaving moths with a potential mechanism for adjusting thermoregulation. This idea has been proposed several times [41–43], but such functionality could not be confirmed [24]. Nonetheless, microstructures can alter infrared spectral emissivity [44] as well as angular emissivity [45]. In addition, a measure of surface roughness, which determines the specularity, depends on the wavelength of light in question [46]. Infrared waves do not resolve the smallest structures and also experience a steeper gradient of refractive index [39] within a structured surface, with the result that specularity increases towards infrared wavelengths [47–49].

It is conventional wisdom that picometre-sized atoms and molecules, too small to resolve with microscopy, can be imaged with spectral imaging [50]. Similarly, submicron-dominant spatial frequencies can be deduced from coherent tissue colours [51] or resonant thin films [39]. Even incoherent reflectance can, in some situations, reveal microscopic features, thus circumventing the imaging resolution criterion [52,53]. In this study, we demonstrate how information concerning lateral microscopic features on moth wings might be retrievable over long distances.

Here, we investigate the coherent and incoherent short-wave infrared properties of the ventral and dorsal sides of wings in 82 individuals representing 26 species of moths. We demonstrate that moth wings generally become glossier towards the infrared but that both surface roughness and the steepness of this increase consistently differ between species. We provide simple physical models and parameterization for both coherent and incoherent scattering from both the ventral and dorsal sides of moth wings. We investigate the diversity that is apparent among moths and explain the potential for remote species identification by laser radar, discussing optimal bands for specificity. Ultimately, we demonstrate how this increased flatness at infrared wavelengths can increase information richness in a lidar identification scenario.

## 2. Results

### 2.1. Infrared properties of moth wings

**Figure 1.**
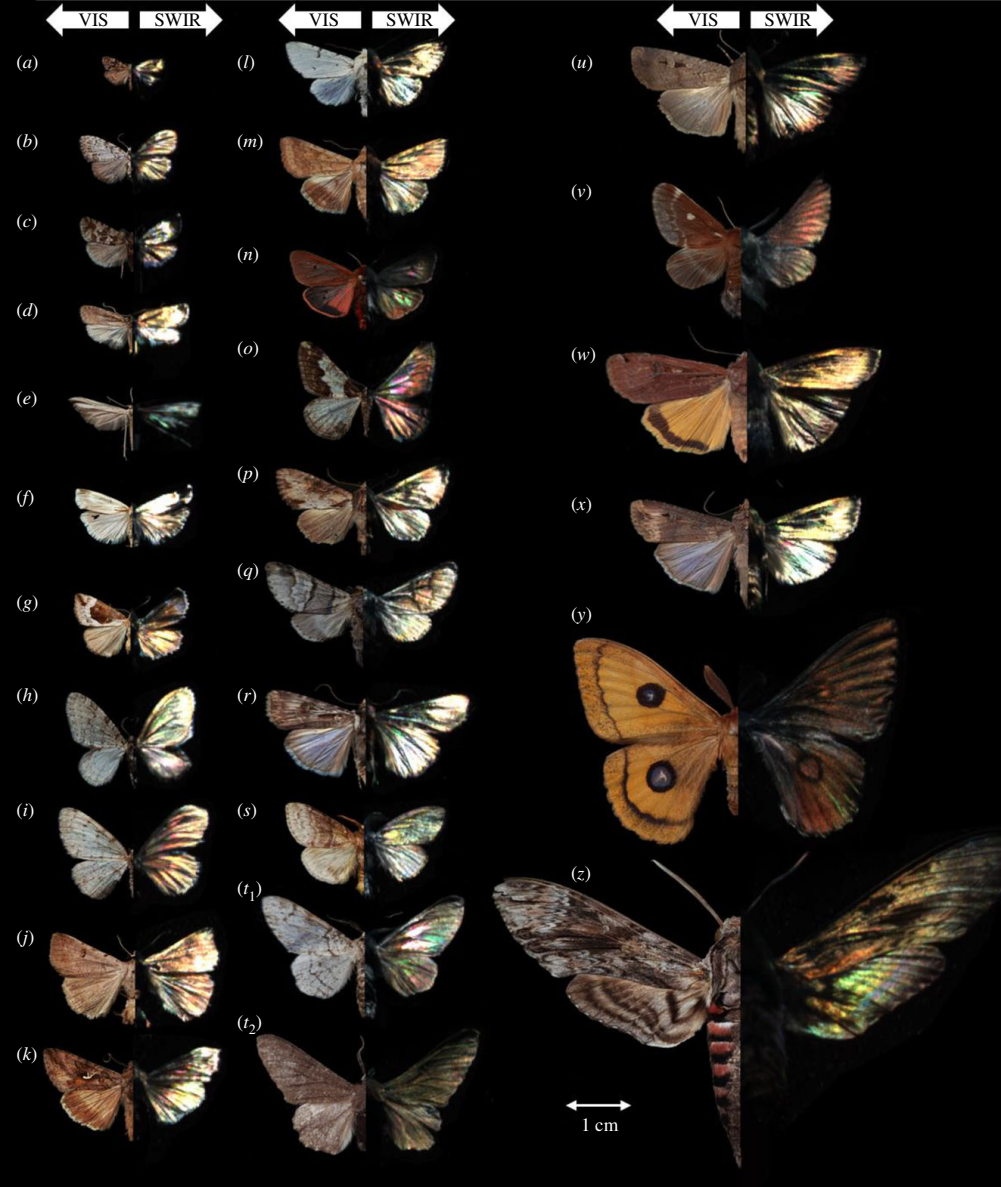
Twenty-six moth species examined in the study are presented in both true- and false-colour images. The left halves of the moths are shown in true-colour RGB (VIS) images taken with a commercial camera with diffuse illumination. The right halves of the moths are shown in false-colour SWIR images scanned with a hyperspectral camera in specular illumination (band choices: blue at 1250 nm, green at 1700 nm and red at 2300 nm) with a gain of 40%. Species of moths: (*a*) *Tebenna bjerkandrella,* (*b*) *Meganola strigula,* (*c*) *Phiaris schulziana,* (*d*) *Agonopterix ciliella,* (*e*) *Emmelina monodactyla,* (*f*) *Crambus perlella,* (*g*) *Hypena crassalis,* (*h*) *Trichopteryx carpinata,* (*i*) *Aethalura punctulata*, (*j*) *Lygephila craccae,* (*k*) *Autographa gamma,* (*l*) *Acronicta leporina,* (*m*) *Helicoverpa armigera,* (*n*) *Phragmatobia fuliginosa,* (*o*) *Agriopis leucophaearia,* (*p*) *Apamea crenata,* (*q*) *Furcula bifida,* (*r*) *Agrotis munda,* (*s*) *Calliteara pudibunda,* (*t*_1_,*t*_2_) *Biston betularia,* (*u*) *Agrotis infusa,* (*v*) *Eriogaster lanestris,* (*w*) *Noctua pronuba,* (*x*) *Agrotis ipsilon,* (*y*) *Aglia tau* and (*z*) *Agrius convolvuli*.

Hyperspectral imaging was carried out on 82 moth specimens from 26 species. Specular images of each species are presented as true-colour-visible (VIS) images and false-coloured short-wavelength infrared (SWIR) images in figure 1. Many moth wings display glossiness in the SWIR image, and some even display structural colour (figure 1*n*,*o*,*t_1_*,*x*; note that gain is reduced for specular images to avoid saturation).

Hyperspectral imaging was performed for all specimens under three different illumination configurations to study coherent, incoherent and diffuse reflectance from the dorsal and ventral sides of the moth wing (see the illustration in the electronic supplementary material, figure S1). We evaluated the effective reflectance spectra integrated over the entire moth wing surface, as these are properties that can be retrieved by lidar over far distances. These effective reflectance spectra were then parameterized by several spectral reflectance models. Our models rely on parameters in SI units for intercomparability between studies (see electronic supplementary material, tables S1–S6). Co-polarized effective reflectance of 25 out of 26 species could be explained by a long-pass function model (but seven species were out of our instrument spectral range) and de-polarized and diffused reflectance of 26 out of 26 species could be explained by a de-polarized/diffuse reflectance model. Apart from spectral parameterization, we also describe and parametrize the depolarization features of moth wings, which is another aspect that can be retrieved remotely.

The probability of correct identification of moth species based on the parametrized values was estimated for ventral remote identification. There are nine parameters for co-polarized, de-polarized and diffuse classification (see electronic supplementary material, tables S2, S4 and S6). For each species, we produced 10^4^ synthetic data points with the corresponding median and interquartile range (IQR) for each parameter; the synthetic data were then fed into naive Bayes classifiers. The covariance between parameters was not included. The parameter overlap was estimated between species and visualized by a standard confusion matrix (see electronic supplementary material, figures S32–S37). Within the selection, several species could be distinguished with high accuracies in the range of 80–90%, even with just three parameters from either diffuse, co-polarized or de-polarized measurements (see electronic supplementary material, figures S32–S34). However, with only three parameters, the species with large parameter overlap are also the species with an asymmetric IQR caused by outliers due to wing alignments. The success of identification drastically improves when six parameters are included; for example, co-polarized parameters in conjunction with de-polarized or diffuse properties (see electronic supplementary material, figures S35 and S36). When all nine ventral parameters from co-polarized, de-polarized and diffused classification were used to identify species, the system has nearly 100% accuracy for most of the species, as shown in electronic supplementary material, figure S37. From a lidar design perspective, acquiring all nine parameters, however, implies a rather complex system. The three co-polarized parameters imply that coherent moth wing properties could be captured by a triple-band lidar system. In addition, such a system would simultaneously be able to capture the diffuse properties in between the coherent flashes in the wingbeat cycle. Band placement would need to include short bands for melanin, a long band where the infrared flatness is captured and an intermediate band in relation to the steepness coefficient, *α*.

**Figure 2.**
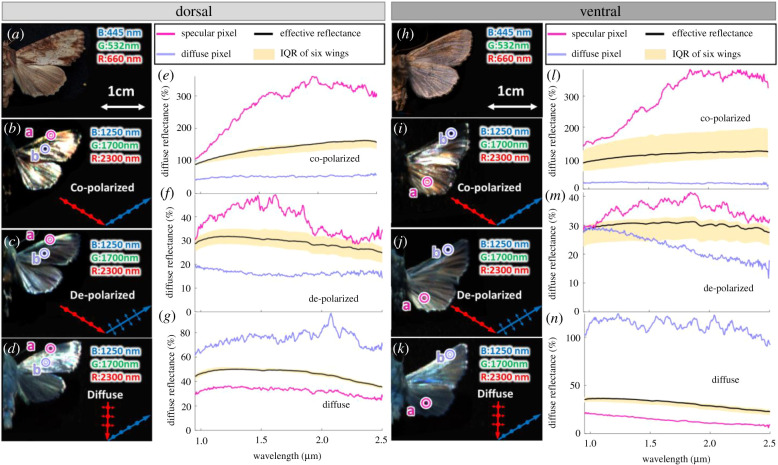
The reflectance of a specular pixel (pink circle labelled ‘a’), a diffuse pixel (mauve circle labelled ‘b’) and entire wing pixels from the dorsal and ventral sides of the moth *A. crenata*. *Red* and *blue arrows* (in (*b–d*)*,* (*i–k*)) indicate the illumination and detection configurations. In both (*b*,*i*), incident and received signals are horizontally polarized (*dots* on the *arrow* indicate that the polarization direction is perpendicular to the plane of the paper). In (*c*,*j*), the incident light is horizontally polarized, and the received signal is vertically polarized (the polarization direction is parallel to the plane of the paper). In (*g,n*), the incoming light is unpolarized and coincides with the surface normal, and the received signal is horizontally polarized. The *yellow area* in (*e–g*) and (*l–n*) highlights the effective reflectance (IQR spread of six individual wings of the same species).

Examples of effective reflectance spectra under coherent co-polarized, incoherent de-polarized and diffuse illumination are shown for the moth *Apamea crenata* in figure 2. Apart from one species (*Agriopis leucophaearia*) displaying a fringed spectrum, the remaining moths display similar spectra to those shown in figure 2 but with distinct magnitudes, cut-on wavelengths and spectral steepness (see electronic supplementary material, tables S1 and S2). In addition, the cut-on wavelength of seven species exceeded our instrument range; thus, the cut-on wavelength could not be determined. Coherent co-polarized spectra from both the dorsal and ventral sides of the moth wings (figure 2*e*,*l*, solid black line) exceed 100% diffuse reflectance. Co-polarized coherent reflection, or the specular reflectance lobe from glossy samples, has a degree of dependence on the incidence angle and predominantly contributes to the signal in the specular imaging mode. By contrast, diffuse targets produce a Lambertian reflectance lobe where a smaller portion of the scattered light reaches the camera (see electronic supplementary material, figure S1*B*). All spectra were calibrated to a diffuse Lambertian reference, and strong specular co-polarized light reflectance exceeded Lambertian white. The coherent reflectance increases towards longer wavelengths (figure 2*e,l*), while the incoherent reflectance decreases with wavelength (figure 2*f,m*). Both these effects could be explained by the fact that long-wavelength infrared photons experience rough surfaces flatter than their short-wavelength counterparts. Thus, the rough surface features on moth wings cannot be resolved at the longer wavelengths. Consequently, wings display smoothness and glossiness towards infrared. The inverse phenomenon can also be observed in figure 2*g,n,* where the moth wings were under diffuse angle illumination (illustrated in the electronic supplementary material, figure S1*A,C*). As the moth wing surface appears smoother at longer wavelengths, the diffuse lobe gradually morphs into an elongated specular lobe along the surface normal, implying that less light reaches the camera (see light lobe shape changes from electronic supplementary material, figure S1*C–*S1*A*). The spectral increase in coherent co-polarized reflectance in figure 2*e,l* and the decrease in the incoherent de-polarized reflectance in figure 2*f,m* at a longer wavelength indicate that mainly specular-reflected photons remember their original polarization state.

Interestingly, a large proportion of the investigated species display consistently distinguishable parameters in both the spectral and polarization domains. Such a result considerably raises the prospects for remote identification of moths. To exploit the increased flatness of moth wings in the infrared regime to assist with insect species identification in lidar data, we need to understand what those extracted physical parameters in the electronic supplementary material, tables S1–S8 represent and how they are correlated to species and wing surface periodic nanostructures. All physical parameters in the electronic supplementary material, tables S1–S8 are plotted as scatter plots and are shown in the electronic supplementary material, figures S2–S21 for coherent, incoherent, diffuse and degree of linear polarization (DoLP) measurements. The data points are the median of multiple specimens of each species, and the indicated spread is by the IQR within the same species. As moth wings become glossier towards infrared wavelengths, their physical parameters consistently differ between species (see electronic supplementary material, figures S2 and S3) and, for each species, these parameters generally do not overlap with those of the majority of the remaining investigated species. This tendency also prevails in scatter plots of the rest of the optical parameter spaces (see electronic supplementary material, figures S3–S21 for both ventral and dorsal sides).

To remotely identify freely flying moths, their ventral infrared properties could be retrieved by entomological lidar [54] in vertical mode. Such lidars can be implemented with polarization sensitivity [55] and/or with dual band in the short-wave infrared [56]. Wings make the largest contribution to the backscattering, and most of the wing signal is coherent and specular. Therefore, the optical properties of moth wings from the ventral side under coherent co-polarized illumination contain the information that is useful for developing a moth-identifying vertical lidar. There are a few sources of environmental noise that may interfere with moth identification. Daylight suppression is generally a challenge in multiband atmospheric lidar but is less of an issue for monitoring nocturnal moths. Atmospheric turbulence can cause low-frequency noise that can be confused with the moth wingbeat, but this mainly occurs during the daytime. Water vapour can attenuate several bands within the infrared spectral range. On shorter ranges, e.g. a kilometer this attenuation is less severe than through the entire atmosphere.

As alluded to above, the coherent co-polarized wing reflectance can be explained by a long-pass function with three parameters: *R*_long_, the maximum reflectance for long wavelengths; *λ*_0_, the surface roughness (the cut-on wavelength of the long-pass function in micrometres at which the surface turns from diffuse to specular); and *α*, the long-pass steepness (like filter order, this is unitless). For remote lidar sensing applications, *R*_long_ will be challenging to estimate because of the orientation of the wing as well as the position in the beam. Therefore, the surface roughness *λ*_0_ and the steepness *α* are better candidates for remote identification. Since the spectral features *λ*_0_ and *α* could not be associated with molecular absorption (i.e. by melanin and chitin), they necessarily relate to the microstructures of moth wings and could thus be used to differentiate species through remote microscopy. Specular wing properties are known to produce rich harmonic spectra which can be sensed remotely [57]. All spectral and polarimetry specular reflectance parameters from the moth ventral side are provided in electronic supplementary material, table S2, and a scatter plot of surface roughness *λ*_0_ versus steepness *α* is presented in electronic supplementary material, figure S3.

**Figure 3.**
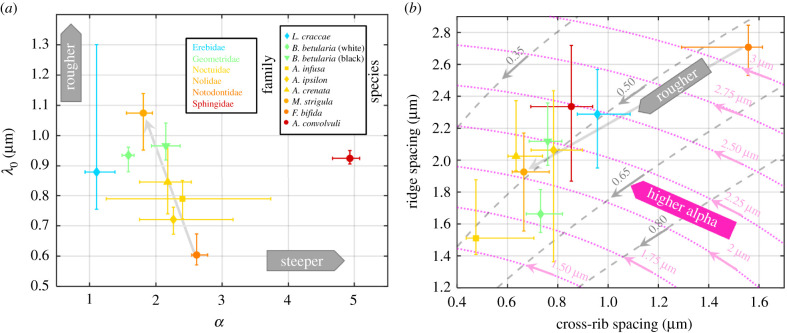
Correlation of the physical model parameters to moth wing scale nanostructures. (*a*) Scatter plot of the physical parameters, surface roughness *λ*_0_ versus steepness *α*, obtained from ventral specular reflectance spectra. (*b*) Scatter plot of ridge and cross-rib spacing from the SEM scans of moth wing scales. Bars indicate ¼ and ¾ quartiles within species variation. Grey auxiliary lines are drawn to illustrate power relations and the pink auxiliary lines are used to describe isotropic curves of periodicity in micrometres.

**Figure 4.**
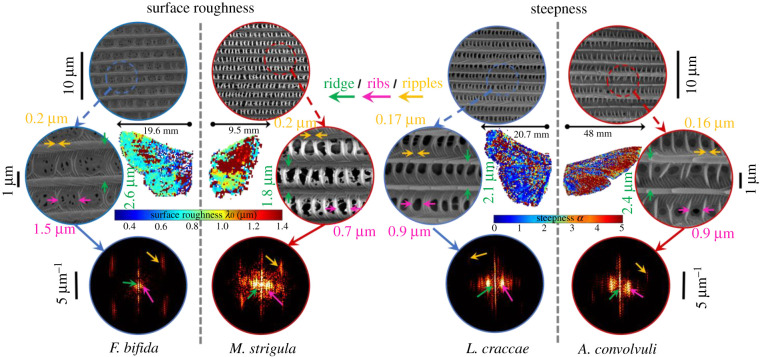
False-colour image of extreme cases of moth wings in four species in terms of surface roughness and steepness difference (*central images*) with their corresponding SEM images. Wing images are colour coded to present the pixel-wise surface roughness or steepness distribution across the entire wing. Magnified SEM images with *arrows* highlighting example spacings between ridges (*green arrows*), cross-ribs (*pink arrows*) and ripples (*yellow arrows*) for each species and where they are located in the fast Fourier transform images (*lower row*).

Eight species (one species with two subspecies) with extreme values of surface roughness *λ*_0_ and steepness *α* (electronic supplementary material, figure S3) are chosen and highlighted in figure 3*a*. All eight chosen species' wing surface microstructures were investigated with scanning electron microscopy (SEM) as part of this study. Two extreme cases from eight species in surface roughness and two other extreme cases in steepness are shown in figure 4 as colour-coded wing images, where *λ*_0_ and *α* are calculated on a pixel level. The figures map out the surface roughness and steepness changes among the wings and provide a sense of which wing region has the greatest influence on the value we parametrized across the entire wing.

### 2.2. Electron microscopy of wing scale structures

The periodicity of ventral nanostructures was investigated using SEM. Moth wings are covered in scales (both ventrally and dorsally), which exhibit a periodic two-dimensional pattern on the scale surface. These structures vary in periodicity, with the coarsest feature being the ridges parallel to the scale's main growth axis. Perpendicular to the ridges, a series of more finely spaced cross-ribs can be observed, which are sometimes intersected by ripples. To quantitively assess these nanostructures, spatial frequencies of ridges, cross-ribs and ripples were measured from the SEM images to show the relationship between the different nanostructures and their spatial frequency content (figure 4). Comparing the eight extreme species, there were differences in nanostructures both qualitatively (e.g. the morphology of ridges) and quantitively (spatial frequencies); however, the orientation of scales across the wing surface was the same for all species.

The spatial periodicities of ridges and cross-ribs from the ventral wing surface in all examined species are displayed in figure 3*b*, assigning each moth a distinct position in this parameter space. Notably, periodicity or ripples did not differ significantly among species, and ripple periodicity did not scale with the ridge and rib periodicity. Most species do not overlap with the majority of the other species. Moths of the family Noctuidae and *F. bifida* (Notodontidae) account for the extreme values of *λ*_0_, yet the within-species spread is consistent between them, reflecting their close evolutionary relatedness and thus causing them to deviate from the other species. Although the morphology differed significantly, the ridge and cross-rib periodicities resulted in a few overlapping species (figure 3*b*). The ripple spacing had a median value of 0.17 µm for all species. This might suggest that the ripples could relate to a molecular preference of the chitin polymer plane thickness [58]. Therefore, ripples could be building blocks of the wing surface structure, unrelated to both ridge and cross-rib periodicity. If this is confirmed in later studies, it would imply a severe discretization of lepidopteran microstructures with large implications for both evolution and remote identification. The correlation between the periodicities of ridges, ribs and ripples is presented in the electronic supplementary material, table S9. In particular, we found a correlation of 97% in the relation *d_Ridge_*/*d_Ripple_* = 5.5 (*d_Rib_*/*d_Ripple_*)^½^ among examined species. This result is used in figure 3*b* to describe the aspect ratio between ridge and ribs with a main axis having a square-root power relation (½, solid grey line in figure 3*b*).

### 2.3. Correlation between the spectral properties and moth wing nanostructure

The surface roughness *λ*_0_ and steepness *α* are parameters that have the least sensitivity to the wing orientation and are therefore the best candidates for identifying moth species. Since these relate to the surface microstructure, we related them to the lateral periodicities observed with electron microscopy across the scale surface. The spectral long-pass function can be likened to a road coarsely paved with cobblestones (stone size of *λ*_0_) being irradiated by ping-pong-ball-sized photons of short wavelength *λ* (i.e. *λ* *<* *λ*_0_). These photons would bounce off the road in random directions, whereas basketball-sized photons (*λ* *>* *λ*_0_) would bounce with the memory of the incident angle. Surprisingly, we find that the species with the coarsest lateral periodicity, *F. bifida*, displays the lowest *λ*_0_ value. Reciprocally, we find the finest lateral periodicities in *M. stigula*, which also has the largest *λ*_0_ value (figure 3). Our interpretation is that the distribution of surface normal across the scales has a reduced spread for the coarse lateral periodicities. This could occur in a situation where the lateral periodicities scale but the depth profiles remain constant. Unfortunately, our SEM images do not yield depth profiles.

The ridge–rib aspect ratio (along the isotropic pink lines in figure 3*b*) is somewhat related to the steepness parameter, *α*. For example, *B. betularia* (black) and *A. convolvuli* are both above the main square-root axis while *B. betularia* (white) and *L. craccae* are below it. However, both *A. convolvuli* and *L. craccae* display intermediate *λ*_0_ values as well as intermediate lateral periodicities (from the SEM measurements). The black and white morphs of *B. betularia* are generally thought to differ only in melanization. Interestingly, both the structural steepness parameter *α* and the SEM images (which are blind to melanization) confirm that the scale microstructure differs significantly between the two morphs of this single species. We emphasize that there are cases where morphs and sexes differ, not only in coloration but also in the microstructure. In analogy, previous lidar studies of mosquitoes' wingbeats demonstrate high sex discrimination, whereas species can be more challenging to differentiate [54]. The expectations should thus be that differences in both coloration and microstructure only partly reflect the allocation in the phylogenetic tree and that groups and phenotypes could also be explained by other ecological factors than genetic heritage.

It is evident that the specular spectral properties *λ*_0_ and *α* are associated with microscopic features in the SEM images. It is also evident that the ridge and rib spacing alone are not enough to precisely predict the spectral properties. Knowledge of the depth profiles of moth wing scales is likely to improve spectral prediction.

### 2.4. Implications for remote sensing of freely flying moths

**Figure 5.**
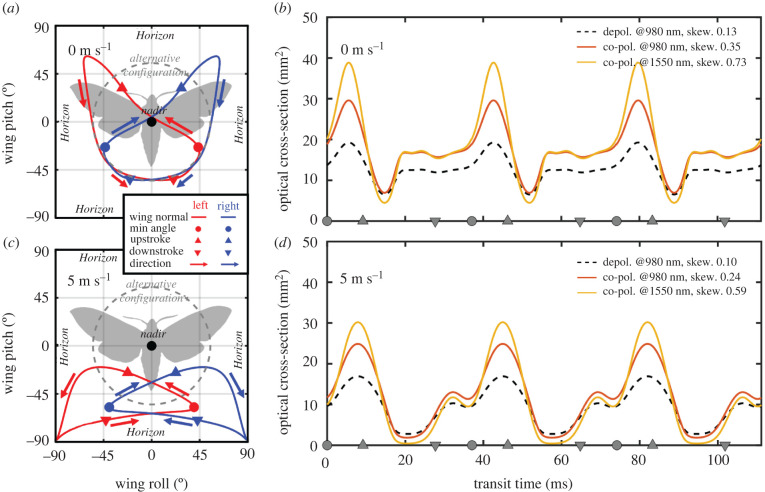
Simulated moth signals with a vertical lidar detection scheme. (*a,c*) Wing surface normal trajectory of a hawkmoth at two flight speeds. When the normal approaches nadir, the wings would produce a ‘flash’ in the vertical lidar signal. (*b,d*) Modelled temporal wingbeat cross-section signal from zenith. Coherent properties increase with wavelength, as does the waveform skewness.

With the knowledge we have gained regarding specular reflectance from moth wings, we fed the parameters of the hawk moth *A. convolvuli* into a BRDF. This model can vary continuously from diffuse Lambertian reflectance to specular reflectance. We then combined this reflectance model with the wing motion angles from a previous study on hawk moths [29]. However, such a model was constructed exclusively for Sphingidae; also fore- and hindwings of some species can conform to a wavy surface rather than a plane during flight. The simulation was intended only as an example to demonstrate the effect of infrared specularity on the scattered waveform. The wing pitch and roll angles during one wingbeat are presented in figure 5*a,c*. We consider the 180° backscattered light from a vertical lidar and multiply the reflectance by the area of the projected wing shapes (figure 5*b,d*). Accordingly, we present the expected lidar waveform signals from flying hawk moths at two flight speeds. The dashed de-polarized diffuse Lambertian signal directly represents the projected wing area at zenith observation. The co-polarized, coherent and specular signal, on the other hand, displays a moderate flash at the instant when the wing surface normal approaches nadir (during upstrokes). This effect increases with a longer wavelength as the wing becomes glossier. At flight speeds of 5 ms^−1^, the wing surface normally approaches horizontal, and the flash could even occur in horizontal lidar observation mode. Alternatively, the likelihood of observing flashes could be maximized by observing at 56° from zenith. The last two detection modes would, however, be highly sensitive to the flight direction. We conclude that infrared specular properties have a large impact on the wingbeat waveforms and are thus retrievable from far distances. The specular properties could thus be assessed by (i) a polarization-sensitive lidar, (ii) a dual-band lidar, or (iii) simply calculating the waveform skewness (see caption of figure 5*b,d*). Since skewness is unitless, this approach would pose a highly robust solution that is insensitive to instrument calibration.

## 3. Discussion

We have shown that moth wings become glossier towards the SWIR, that is, specularity increases with wavelength, a fact that is also reflected by their polarimetric properties. We have proposed and demonstrated reflectance models that can effectively parametrize the coherent, incoherent and diffuse effective reflectance from the dorsal and ventral surfaces of moth wings in the near-infrared (NIR)-SWIR region. Reflectance spectra from the wings of 82 specimens of 26 moth species (one species with two subspecies) are reduced to 11 non-redundant parameters (see electronic supplementary material, figure S22) in SI units for reproducibility. These spectral and polarimetric parameters differ significantly between species, a promising result for remote identification of freely flying moths with optical sensors.

We validated the idea of remote microscopy by correlating the deduced parameters from the infrared reflectance with spatial periodicities deduced in SEM scans of the ventral wing surface. Species with significantly distinct spectral parameters also showed significantly different microstructure periodicities on their wing scales, although in a non-intuitive manner. Species with rougher wing surfaces and becoming shiny at longer wavelengths had smaller spacing between ridges and cross-ribs. We speculate that a more accurate relationship between spectral properties and microstructures could be accomplished with additional measurements of the depth profiles of wing scale surfaces (using SEM). Morphological measurement precision could also be increased in future studies with additional techniques, such as micro-computed tomography.

The majority of moth species were chosen based on their abundance in the southern Swedish province of Skåne, generally with three replicates for each species to estimate variation. However, for some families, we only had one species available. Therefore, when we parameterized all reflectances and analysed how distinct each species is relative to others, we could not conclude if there is any generally applicable trend at the family level. However, the microstructure periodicities [59] and infrared properties [24] in our study compare well with previous reports on both moths and butterflies. Thus, the surface roughness assessment and model could also be applied to butterflies. Within our spectral range, we could not parametrize coherent reflectance for seven of our moth species, presumably because they had a too coarse microstructure. Further research should include more species in the same family and use a spectral system that can identify new extreme values by employing instruments in the 3–5 μm range.

By using the knowledge gained by modelling the reflectance of a hawkmoth and combining this with the wing dynamics from a previous study [29], we could simulate entomological lidar signals, and various bands and polarizations, for the purpose of remote identification of freely flying moths. Indeed, the coherent specular phenomena described in this study have a large impact on the resulting lidar signals. The infrared glossiness can be retrieved differentially with a dual-band lidar [56], through co-polarization- and de-polarization-sensitive lidar [55], or simply by calculating the waveform skewness. We thus can expect that numerous moth species can be differentiated remotely by retrieving reflected optical signals that are directly related to the microstructures of their wing scales.

## 4. Material and methods

### 4.1. Lepidoptera specimens

We included 82 individuals of 26 moth species from 13 different families representing both macro- and micromoths, provided by the Lund University Biological Museum (figure 1). As examined species show no sexual dimorphism, we do not expect any difference between males and females in the spectral and structural signatures. Most species were selected based on their abundance in the Skåne region of Sweden [60] (unpublished data based on museum material in the Biological Museum, Lund University), except for two common Australian species. The Swedish moth species included were *Agonopterix. ciliella* (Depressariidae); *Agrius convolvuli* (Sphingidae); *Apamea crenata, Autographa gamma, Agrotis ipsilon, Acronicta leporina, Helicoverpa armigera, Noctua pronuba* (Noctuidae); *Agriopis leucophaearia, Aethalura punctulata, Biston betularia carbonaria* (black), *Biston betularia typica* (white), *Trichopteryx* *carpinata* (Geometridae); *Aglia tau* (Saturniidae); *Crambus perlella* (Crambidae); *Calliteara pudibunda, Hypena crassalis, Lygephila craccae, Phragmatobia fuliginosa* (Erebidae); *Eriogaster lanestris* (Lasiocampidae); *Emmelina monodactyla* (Pterophoridae)*; Furcula bifida* (Notodontidae); *Meganola strigula* (Nolidae); *Phiaris schulziana* (Tortricidae); and *Tebenna bjerkandrella* (Choreutidae). Two Australasian noctuids were also included in the study to increase the number of species in the genus *Agrotis* (*Agrotis infusa* and *Agrotis munda*) to test whether lidar has the potential to distinguish species within a tight taxonomic group.

Moth wings were spread and set in a horizontal plane by a skilled expert to minimize damage or wearing to the wing scales during handling. Estimation of within-species variation was allowed by at least three replicates of each species variance (except *A. munda*, a single specimen) and measurements were taken from both the left and right wings for both the dorsal and ventral sides of the body. Even if a wing should have minor damage where scales are not intact, it has minimal influence on the reported median value. In regards to the age of the specimen, a study on the wing interference pattern displayed on transparent insect wings is stable even on 100-year-old dry museum specimens [61]. Also, the nanostructures in butterflies, which produce structural colours in the visible range, are generally considered to be stable in museum collections. Our presumption is thus that the chitin microstructure on dry moth scales should also remain stable over time.

### 4.2. Hyperspectral imaging

The NIR-SWIR optical properties of moth wings were studied using a push-broom hyperspectral camera [62] (HySpex; Norsk Elektro Optikk AS, Norway). Two 150 W broadband halogen lamps were used sequentially to illuminate the sample, and two ultra-broadband polarizers [63] (Meadowlark Optics, USA) were used for polarimetric analysis. All specimens were mounted on black neoprene foam sheets to minimize the reflection from the background. The hyperspectral camera was operated with an objective with an aperture of ø25.4 mm and a working distance of 30 cm. The push-broom scan generates a three-dimensional (*x*, *y*, *λ*) hyperspectral image cube, where the *λ* includes 288 spectral bands ranging from 0.95 µm to 2.5 µm and a spatial resolution of 240 µm. All recorded hyperspectral data were calibrated to a target with a 50% diffuse reflectance standard (Spectralon^®^). The angular light distribution was captured by imaging silicon nitride spheres [64].

The hyperspectral imaging was repeated with three different configurations of illumination (electronic supplementary material, figure S1). The configurations were as follows. (i) Co-polarized specular illumination (with horizontal linear polarizers on both illumination and camera). The illumination and camera axis were at ±56° to surface normal. (ii) De-polarized illumination (with a horizontal polarizer on the light source and a vertical polarizer on the camera). Again the illumination and camera axis were at ±56° to surface normal. (iii) Diffuse illumination (illuminating with unpolarized light and capturing the horizontally polarized light). In this case, the illumination coincided with the surface normal, whereas the camera remained at 56°. Examples of reflectance spectra from a specular pixel, a diffuse pixel and the entire wing of *A. crenata* are shown in figure 2.

### 4.3. Parameterization of specular reflectance (co-polarized model)

We generally observed that the specular reflectance increased and reached a plateau towards infrared (see example in figure 2*e,l*). Thus, we applied a long-pass function known from electrical and photonic engineering:

4.1 $$R=R_{\mathrm{long}}\frac{{\left(\lambda /\lambda_{0}\right)}^{\alpha }}{1+{\left(\lambda /\lambda_{0}\right)}^{\alpha }}.$$

In this model, *R*_long_ is the asymptotic maximum reflectance value, *λ*_0_ is the cut-on wavelength or surface roughness and *α* is the slope steepness of the spectrum. All parameters are provided in the electronic supplementary material, tables S1 and S2. For the majority (19) of specimen explanations, grades (*R*^2^_adj_) exceed 98%. For seven species, reflectance continuously increased towards infrared without reaching a plateau within our spectral range. This does not imply that our model is inaccurate, but rather that the surface is too rough to assess with the spectral range of our instrument (0.9–2.5 µm). This also prevents us from identifying both *R*_long_ and *λ*_0_. The last remaining species (*A. leucophaearia*) displayed a fringy spectrum that does not comply with equation (4.1).

### 4.4. Parameterization of diffuse reflectance (de-polarized and diffuse model)

The majority of diffuse reflectance spectra decrease towards both short and long wavelengths (figure 2*f*,*g*,*m*,*n*). The decrease towards a short wavelength below 1.2 µm can be explained by eumelanin absorption [65]. The decrease in diffuse reflectance towards long wavelengths is primarily due to transmission losses owing to diffuse photon migration and random walks [66] and the increasingly specular surface (thus not contributing in diffuse mode). The mean free path increases with wavelength, and so does the chance of light escaping on the back side of the wing (and thus not contributing to reflectance). A model was developed that was inspired by the Beer–Lambert law:

4.2 $$R_{\mathrm{diff}}\approx R_{\mathrm{depol}}=R_{0}\cdot e^{-\mu_{a}\ell_{\mathrm{mel}}-\mu_{T}\lambda },$$

where *R*_0_ is the maximum diffuse reflectance, *ℓ*_mel_ is the equivalent pathlength in pure melanin and *µ*_T_ is a slope coefficient arising because of transmittance. *µ*_a_ is pure eumelanin absorption and is also wavelength dependent [65]:

4.3 $$\mu_{a}=\varepsilon (\lambda )\cdot C=\varepsilon_{0}e^{-(\lambda /\lambda_{m})}\cdot C,\;\varepsilon (\lambda )=\varepsilon_{0}e^{-(\lambda /\lambda_{m})},$$

where *ɛ*_0_ = 2.45 µm^−1^ M^−1^, *λ*_m_ = 0.175 µm and *C* = 5.65M

Increased melanin pathlength, *ℓ*_mel_, attenuates the shorter part of the reflectance spectra. An increase in the slope coefficient *µ*_T_ will attenuate the longer parts of the reflectance. The parameters and explanation grades are provided in electronic supplementary material, tables S3–S6.

### 4.5. Parameterization of degree of linear polarization spectrum

To investigate the feasibility of polarimetric identification of moth wings, the spectral depolarization was investigated by calculating the DoLP:

4.4 $$\mathrm{DoLP}=\frac{I_{\mathrm{co}}}{I_{\mathrm{co}}+I_{\mathrm{de}}}=\frac{1+e^{-{\left(\lambda_{p}/\lambda \right)}^{\gamma }}}{2}.$$

Note that there are several definitions of DoLP in the literature. The one above gives 100% for entirely co-polarized reflectance and 50% for entirely randomized reflectance. In equation (4.4), *I*_co_ and *I*_de_ are co-polarized and de-polarized reflected intensities, respectively, *λ*_p_ is the wavelength where wings become co-polarized. The spectral dependence of DoLP is given by *γ*. In summary, a high *λ*_p_ implies a diffuse wing, and a high *γ* implies that DoLP increases with wavelength. All parameters are documented in electronic supplementary material, Tables S7 and S8, where *R*^2^ indicates the fitting quality.

### 4.6. Scanning electron microscopy

Eight of 26 moth species (one species with two subspecies) were selected to investigate moth wing nanostructures using SEM. The selection was made based on the parametrization results from specular reflectance signals from the moth ventral wings (electronic supplementary material, figure S3). *A. convolvuli, F. bifida, L. craccae* and *M. strigula* were chosen because they represent the extreme values for surface roughness *λ*_0_ and steepness *α*, respectively. *A. crenata* represents the average for both parameters. *A. infusa* and *A. ipsilon* were selected to investigate the variance of nanostructures within the same genus. *B. betularia* was chosen to include another superfamily, the Geometroidea. This species inhabits a polymorphism with two different morphotypes differing in melanization. For each species, one specimen was investigated. Wings were first cut with the help of a stereomicroscope (ZEISS Stemi DV4), and three pieces of each wing were removed. Pieces were about 3 × 3 mm in size and taken from different areas of the wings. Both fore- and hindwings were inspected. The dried wings were carefully glued onto SEM stubs, air-dried and sputter-coated with gold (Cesington 108 auto, 75 s, 20 mA). Imaging was done with a scanning electron microscope (Hitachi SU3500) at 5 kV. For numerical analysis, the SEM images (of resolution 0.025 µm pixel^−1^) were analysed to extract the different lateral spatial frequency components that could correlate with or account for the distinct infrared properties observed.

Each image from the samples was cropped into three windows across the scales. The windows were 20 × 20 µm. The windows were subject to Gaussian apodization, after which the two-dimensional spatial frequency power spectrum was calculated and averaged for the cropping. Three periodic structures were identified, with ridges being the coarsest (approx. 2.0 µm), cross-ribs of intermediate size (approx. 760 nm) and ripples being the finest (approx. 170 nm).

### 4.7. Bidirectional reflectance distribution function for waveform modelling

The reflected light lobe was modelled as a function of incident pitch and roll angles, *θ*, *φ*. The model originated from diffuse Lambertian reflectance, *I* = *I*_0_cos^1/*r*^(*θ*), where *r* represents surface roughness. Here *r* = 1 is a perfect diffusor and *r* = 0 is a perfect mirror. By comparing our hawk moth recordings, we determined *r*_980 nm_ = 0.53 and *r*_1550 nm_ = 0.37 for *A. convolvuli.* To model the dependency of the specular reflectance lobes on the incident light, we introduced a symmetric link function, *F*_link_*(θ,θ*_0_), with the property *F*_link_(*θ, –θ*) = 0° (maximum of the cosine function). This ensures that incident and reflected light angles are opposite. Furthermore, we multiply *θ*_0_ by 1 – *r*; thus, the reflectance lobe of a perfect diffuser coincides with the surface normal and becomes identical to Lambertian reflectance:

4.5 $$\begin{array}{rl} & I(\theta ,\varphi )=I_{0}{\left(\cos(F_{\mathrm{link}}(\theta ,(1-r)\theta_{0}))\cos(F_{\mathrm{link}}(\varphi ,(1-r)\varphi_{0}))\right)}^{1/r},\;\\ & \;r\in \{0\ldots 1\}, \end{array}$$

4.6 $$\begin{array}{rl} & F_{\mathrm{link}}(\theta ,\theta_{0})=90^{\circ }\left({\left(\frac{\theta +90^{\circ }}{180^{\circ }}\right)}^{\frac{-\log(2)}{\log\left(\frac{90^{\circ }-\theta_{0}}{180^{\circ }}\right)}}-{\left(\frac{90^{\circ }-\theta }{180^{\circ }}\right)}^{\frac{-\log(2)}{\log\left(\frac{\theta_{0}+90^{\circ }}{180^{\circ }}\right)}}\right),\\ & \;\theta ,\theta_{0}\in \{-90^{\circ }\ldots +90^{\circ }\} \end{array}$$

4.7 $$\int \int_{-90^{\circ }}^{90^{\circ }}I(\theta ,\;\varphi )\;d\theta d\varphi =1\;,$$

where *I* is the light reflected in the directions *θ* and *φ*. *I*_0_ is a scalar intensity, while *θ*_0_ and *φ*_0_ are the incidence angles.

The BRDF was used to calculate the 180° backscatter from a moth using a vertical lidar. The dynamic wing roll and pitch were adopted from a previous study [29]. The backscattered reflectance during the wingbeat was multiplied by the ventral projected wing area during the wingbeat, and the optical cross-section was obtained as a function of time. The de-polarized signal is modelled with *r* = 1. We note that this model does not consider diffraction and reflectance anisotropy from the grating-like lateral periodicities on the scales. The periodic features on the scales are unlikely to be in a phase across the wing, and diffracted orders are not observable in the zeroth order, as is the case for our specular hyperspectral imaging and/or backscattering in lidar.

## Data Availability

The data are provided in the electronic supplementary material [67].
